# Recent Advances and Future Perspectives on Microfluidic Mix-and-Jet Sample Delivery Devices

**DOI:** 10.3390/mi12050531

**Published:** 2021-05-07

**Authors:** Majid Hejazian, Eugeniu Balaur, Brian Abbey

**Affiliations:** ARC Centre of Excellence in Advanced Molecular Imaging, Department of Chemistry and Physics, La Trobe Institute for Molecular Sciences, La Trobe University, Melbourne, VIC 3086, Australia; m.hejazian@latrobe.edu.au (M.H.); e.balaur@latrobe.edu.au (E.B.)

**Keywords:** microfluidics, micro-mixer, micro-jet, XFEL, molecular imaging, sample delivery

## Abstract

The integration of the Gas Dynamic Virtual Nozzle (GDVN) and microfluidic technologies has proven to be a promising sample delivery solution for biomolecular imaging studies and has the potential to be transformative for a range of applications in physics, biology, and chemistry. Here, we review the recent advances in the emerging field of microfluidic mix-and-jet sample delivery devices for the study of biomolecular reaction dynamics. First, we introduce the key parameters and dimensionless numbers involved in their design and characterisation. Then we critically review the techniques used to fabricate these integrated devices and discuss their advantages and disadvantages. We then summarise the most common experimental methods used for the characterisation of both the mixing and jetting components. Finally, we discuss future perspectives on the emerging field of microfluidic mix-and-jet sample delivery devices. In summary, this review aims to introduce this exciting new topic to the wider microfluidics community and to help guide future research in the field.

## 1. Introduction

The three-dimensional structure determination of biological molecules is a critical step for understanding the dynamics of biological reactions and is essential for rational drug design [1]. The emergence of X-ray Free-Electron Lasers (XFELs) has facilitated the measurement of complex protein structures and the associated dynamics of biomolecular systems with atomic resolution [2]. These experiments require rapid and precise delivery of the liquid sample to the X-ray interaction region in order to capture the structural changes that occur in biomolecules on sub-microsecond to millisecond timescales [3]. The use of microfluidic mix-and-jet devices capable of triggering reactions and delivering liquid samples to the X-ray beam via a free-standing jet has become a reliable technique for solving the structure of biomolecules. The free-standing jet provides a continuous supply of liquid sample solution to the high-intensity X-ray pulses whilst minimising background diffraction noise and radiation damage [4]. Over the past decade, innovative fabrication techniques have led to numerous efficient sample delivery solutions using microfluidic technology capable of both rapid mixing and the creation of a free-standing liquid jet [5]. Additionally, cryogenic Electron Microscopy (cryo-EM) is another well-established experimental technique for studying the structure of biomolecules and their dynamic conformational changes [6]. Microfluidic sample delivery devices have also been employed for pre-mixing and deposition of liquid samples onto cryo-EM grids for time-resolved studies [7].

The Gas Dynamic Virtual Nozzle (GDVN) is currently the most commonly employed method for focusing and accelerating liquid sample streams and creating free-standing liquid micro-jets [8]. Injectors that take advantage of the GDVN principle of flow focusing can be categorised based on their fabrication method, i.e., capillary, lithography-based microfluidics, and 3D printed nozzles. A capillary GDVN usually comprises two co-axial capillaries, e.g., fused silica and ceramic hollow capillaries. The inner capillary carries the liquid sample, and the end of the outer capillary is tapered to further focus the gas flow and create a liquid micro-jet [4]. Lithography-based microfluidics nozzles, which often employ high resolution and replicable lithography techniques in their fabrication, have also recently been of interest. Lithography-based microfluidics technology offers greater flexibility in terms of microchannel geometry design beyond conventional capillary-based approaches, allowing for multiple microfluidic components to be integrated onto a single chip. By implementing this method for microfluidic jetting, the GDVN nozzle is fabricated as one of the features of the lithography-based microfluidics alongside other components, e.g., a micro-mixer, on the same chip.

The recently developed 3D printed microfluidic technology offers a host of advantages over standard methods. It enables the creation of low-cost and rapid prototyping of microfluidic devices with intricate 3D designs, which can be readily adjusted at minimal additional effort [9]. Despite the current fabrication challenges [10,11], 3D printing for microfluidic device fabrication has been rapidly moving toward becoming the dominant microfluidic fabrication method for numerous biochemical and biomedical research projects [12,13,14].

Microfluidic mixers can be classified as either ‘active’ or ‘passive’ mixers. Passive micromixers often use complex channel geometries in order to amplify the chaotic advection effect. Passive micromixers are usually integrated into microfluidic sample delivery devices to rapidly mix the solutions and to trigger a reaction before the mixed solution is delivered via the liquid microjet. The chaotic advection effect in passive micromixers maximises the contact surface for mass transfer between the mixed solutions, and consequently increases the overall mixing efficiency [15]. A variety of different designs for passive microfluidic mixers have been reported in the literature [16,17,18]. For example, Lee et al. [19] have previously systematically reviewed the most common passive micromixer designs and summarised their operational principles and mixing performance. Recently, Raza et al. [18] presented a comparative review based on quantitative analyses of a wide range of different types of passive micromixers, which included looking at their mixing efficiencies, pressure drops, and fabrication costs.

Mix-and-jet microfluidics is an emerging field that has shown a promising capability for implementation as a sample delivery platform for molecular imaging applications. Here, we review the most recent trends in microfluidic jetting, particularly on-chip microfluidics. We first discuss the main parameters for designing the GDVN component. Next, we discuss each of the leading fabrication approaches’ limitations and challenges, namely: capillary, on-chip, and 3D printed microfluidics. We then explore different experimental methods for the characterisation of both free-standing liquid jets and integrated micromixers. Finally, we highlight the future potential and opportunities for microfluidic jetting, specifically in the context of molecular imaging applications.

## 2. Design Considerations

**Figure 1:**
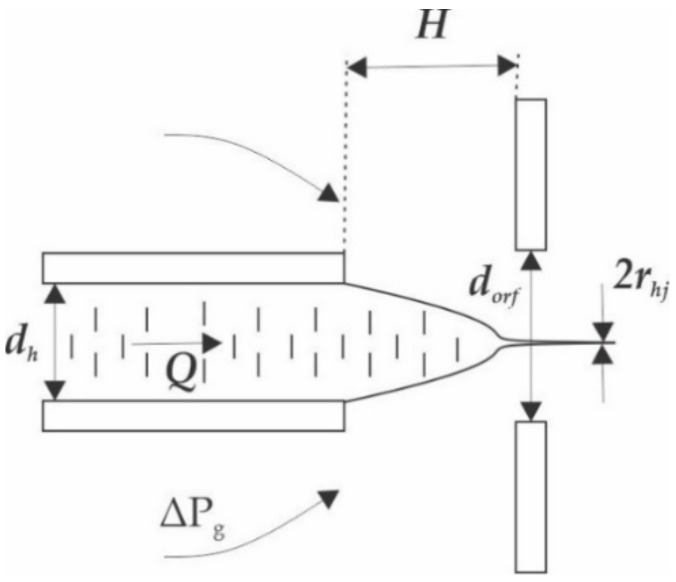
Schematic of the microfluidic nozzle illustrating the main design parameters (reproduced with permission from Reference [21]).

The principle of the GDVN is based on hydrodynamic focusing, which relies on squeezing a continuous fluid stream using a sheath flow, with a different velocity, as depicted in Figure 1. The surrounding sheath fluid, which is injected around the core stream, shapes the core fluid meniscus into a steady micro or nano-jet, which has a smaller size than the outlet microchannel [20]. This section of the review introduces the main design parameters that should be considered when designing GDVN nozzles and passive micro-mixers.

### 2.1. Main Parameters for Nozzle Design

Ganan-Calvo et al. [21,22,23] have described the relative effect of the principle geometrical and flow parameters on jetting stability. Aside from the fluid property parameters, the key parameters that determine the jetting regime with a GDVN nozzle are shown in Figure 1. Liquid with a flow rate of *Q* is injected through the sample microchannel, which has a hydrodynamic diameter of *d_h_*. The liquid meniscus is accelerated via a pressure drop in the gas stream and is hydrodynamically focused in order to form a jet, exiting an orifice with a hydrodynamic diameter of *d_orf_*. The distance from the sample microchannel to the orifice is *H*, and the hydrodynamic radius of the jet is *r_hj_*.

The velocity of the jet can be expressed as

(1) $$V_{jet}=\frac{4Q}{\pi r_{hj}{}^{2}}$$

and the pressure drop of the gas sheath flow is given by

(2) $$∆P_{g}=\frac{\rho_{l}V_{jet}^{2}}{2}$$

where $\rho_{l}$ is the density of the liquid. The Reynolds number (*Re*) is the ratio of the inertial forces versus the viscous forces within the liquid stream and is defined as

(3) $$Re=\frac{\rho_{l}Q}{\pi r_{hj}\mu }$$

where μ is the viscosity of the liquid.

The Weber number (*We*), which is the dimensionless ratio of inertial forces to surface tension forces, is expressed as

(4) $$We=\frac{\rho Q^{2}}{\pi^{2}r_{hj}{}^{3}\sigma }$$

where *σ* is the surface tension of the liquid. The Weber number must be >1 in order to produce a stable jet [24].

Vega et al. [21] experimentally and numerically investigated the effect of these key parameters on jetting stability and mapped the regions of stability and instability using the dimensionless We and Re numbers. From their stability/instability maps, stable jetting tends to occur at relatively higher We and Re numbers, i.e., where the inertial force is dominant. They reported that the transition from unstable to stable regions is mainly determined by the jet dynamics rather than the geometrical parameters. The relative effect of the fluid property parameters, i.e., density, viscosity, and surface tension, are more dominant when working with viscous liquids and very thin liquid jets. Vega et al. also reported that the optimum values for *H* and *Q* increase with increasing orifice diameter.

### 2.2. Main Parameters for Mixer Design

Imaging the dynamics of biomolecules requires sample delivery devices that incorporate micro-mixers capable of efficient mixing with sub-millisecond and millisecond mixing times [25]. The main parameters that are used for the design and evaluation of efficient mixers for integration into mix-and-jet sample delivery devices are the Reynolds number (*Re*), Peclet number (*Pe*), and the mixing efficiency ($\eta_{mixing}$) [19].

The Peclet number is the ratio of convective mass transport rate to the diffusion mass transport rate and is given by:

(5) $$Pe=\frac{ul}{D}$$

where l is the length of the mixing path, and D is the diffusion coefficient. The chaotic advection effect that is induced by the geometry of the passive mixer microchannels leads to local increases in the velocity and, consequently, an increase in the Pe values.

The evaluation of mixing in microchannels is usually achieved by measuring the degree of mixing at different cross-sections within the mixing channel. This can be quantified using the normalised concentration, c^*^, defined as

(6) $$c^{*}=\frac{c-c_{min}}{c_{max}-c_{min}}$$

where c is the concentration of the species in solution, and the subscripts indicate the minimum (*min*) and maximum (*max*) concentration values.

The mixing efficiency ($\eta_{mixing}$) is defined as

(7) $$\eta_{mixing}=1-\;\sqrt{\frac{1}{N}\overset{N}{\underset{i=1}{\sum }}{\left(\frac{c_{i}^{*}-c_{m}^{*}}{c_{i}^{*}}\right)}^{2}}$$

where *N* is the number of sampling points, $c_{i}^{*}$ is the normalised concentration at point *i*, and $c_{m}^{*}$ is the mean normalised concentration.

## 3. Fabrication Methods

There are numerous techniques that are used for the fabrication of integrated mix-and-inject devices. The materials used to fabricate these devices are specially chosen in order to address the key challenges of sample solution compatibility and mechanical stability. Based on the particular fabrication method, the devices can be fabricated with either planar or circular microchannels.

### 3.1. Co-Axial Capillary Devices

The use of co-axial capillary nozzles for the acceleration of a laminar liquid stream to create microscopic free-standing jet flows was first introduced by Ganan-Calvo [22]. Since then, various innovative developments in GDVNs have been reported for their implementation as sample delivery devices for serial femtosecond crystallography (SFX) using XFELs [8].

**Figure 2:**
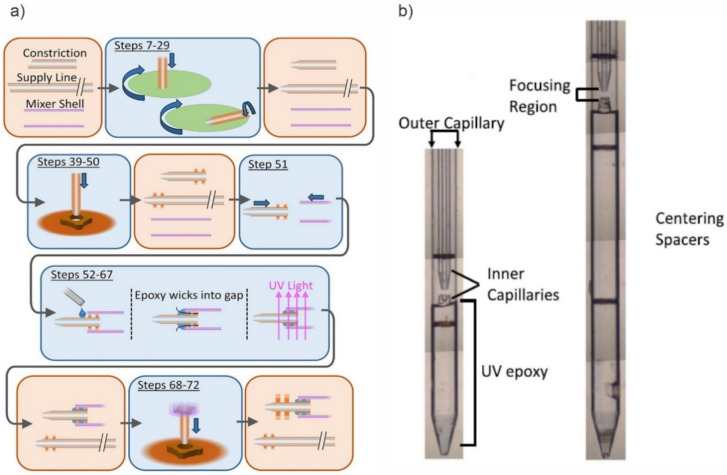
Glass capillary-based GDVN devices. (**a**) overview of the multi-step device fabrication process (reprinted with permission from Reference [28]). (**b**) Optical composite images of the completed devices (reproduced with permission from Reference [27] and Reference [28]).

The capillary devices typically consist of two co-axial glass capillaries, which are co-aligned to form a GDVN. For a typical capillary-based GDVN [26], the liquid sample capillary has an outer diameter of about 50 µm and is tapered at the end. The surrounding co-axial capillary that allows the gas sheath stream to pass has an inner diameter of around 70 µm; with an average liquid sample flowrate of around 10 µL/min, this results in the creation of a liquid jet with a diameter of 4 μm. The conventional fabrication of the GDVN nozzles involves fabricating the individual nozzles by hand, which can result in inconsistent device characteristics. An example of the lengthy 6-step fabrication procedure, shown in Figure 2, was described in detail by Calvey et al. [27,28], which involves multiple flattening, polishing, tapering, and centring steps that require access to custom-designed chucks and jigs. However, using glass capillaries has the significant advantage of high pressure and solution pH resistance and, as a result, has been the most commonly used method for sample delivery for molecular imaging with XFEL [8,29].

Beyerlein et al. [30] introduced an easier and faster manufacturing technique for fabricating capillary nozzles based on ceramic micro-injection moulding. Their method offers a higher resolution (~1 μm) and reproducibility whilst having the advantage of also working at lower flow rates and being stable with respect to high pressures, making them compatible with SFX experiments. Zahoor et al. [31,32] performed comprehensive Computational Fluid Dynamics (CFD) studies of ceramic GVDN’s based on the Volume of Fluid (VOF) and Finite Volume Method (FVM). Their simulation covers a wide parameter space of liquid Reynolds numbers within the ranges of 17–1222, different geometrical parameters, and Weber numbers in the range of 3–320, which can be used for adjustment of the nozzle geometry design and operating conditions for specific liquid samples. However, the ceramic nozzle moulding method also has significant disadvantages, including the high manufacturing cost of the micro-injection moulding tools and misalignment of the inner capillary.

### 3.2. Lithography-Based Microfluidics

**Figure 3:**
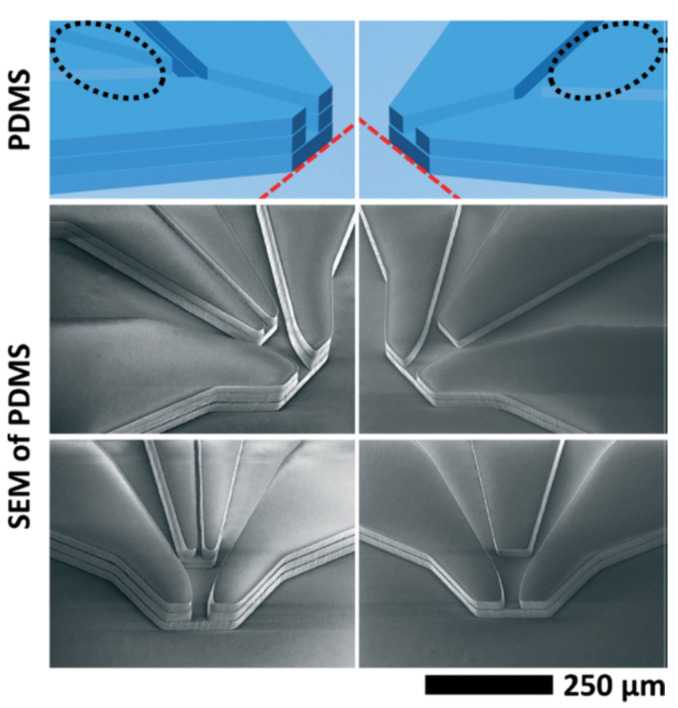
Illustration of the multilayer 3D PDMS microfluidic GDVN devices (reproduced with permission from Reference [33]).

Soft lithography using Polydimethylsiloxane (PDMS) is another conventional method for the fabrication of microfluidic devices. This fabrication approach is fast, high resolution, reproducible, and cost-effective, and allows for the fabrication of high aspect ratio microchannels. Trebbin et al. [33] first reported mix-and-inject microfluidic devices fabricated using a 3-layer bonding PDMS technique, as shown in Figure 3. The technique enables the fabrication of microchannels with different depths, integrating the GDVN nozzle onto a single microfluidic chip, and producing nozzle arrays. Their microfluidic chip could generate liquid jets with diameters ranging between 0.9 and 20 μm. They reported a range of jet diameter, jet length, and the operating conditions under which their devices were able to produce stable jetting under both atmospheric and vacuum conditions.

Feng et al. [34] reported the fabrication of a microfluidic sprayer based on a two-layer PDMS technique for use as an alternative to the conventional pipetting/blotting method of cryo-EM for depositing liquid sample droplets on the EM grid. They reported that by changing the sprayer-grid distance and gas pressure, the ice thickness of the droplets could be controlled. Their proposed micro sprayer has the potential to be implemented for solving the structure of apoferritin using single-particle cryo-EM at high resolution.

Microfluidic GDVN nozzles have also been implemented for the production of microfibers. Zhao et al. [35] used soft lithography with PDMS to fabricate a double flow-focusing nozzle microfluidic chip for the production of microfibers. The double-nozzle technique, using DI-water as a sheath flow, prevents drop formation near the exit of the nozzle and generates a continuous stream of microfibers into the atmosphere. Hofmann et al. [36] implemented the same multilayer PDMS bonding technique used by Trebbin et al. [33] to fabricate a microfluidic nozzle device for the generation of ultrafine fibres. Their approach takes advantage of the GDVN principle, which leads to the creation of a steady and continuous stream of uniform microfibers. Precise control over the microfiber diameter and morphology could be achieved by adjusting the air pressure and solution flow rate.

**Figure 4:**
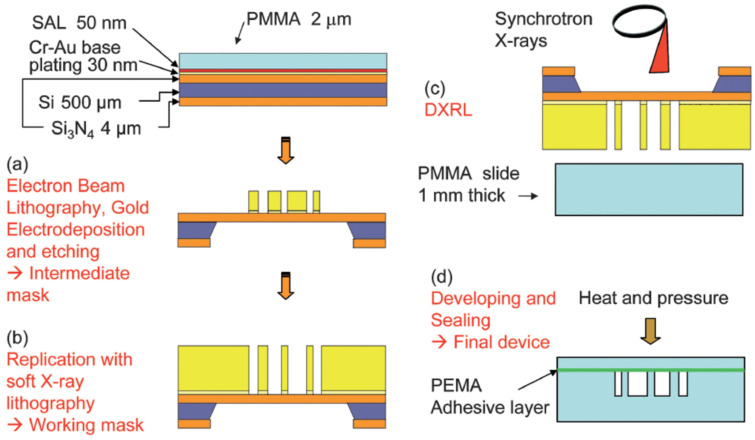
Schematic of the micromixer fabrication process from Marmiroli et al. [37]. (**a**) The production of an intermediate X-ray mask using Electron beam lithography, (**b**) replication of the mask by soft X-ray lithography, (**c**) fabrication of deep micromixer channels on PMMA using Deep X-Ray Lithography (DXRL), (**d**) adhesive bonding of the device. (reproduced with permission from Reference [37]).

Devices made using the soft lithography method with PDMS suffer from low solvent and pressure resistance, which are significant disadvantages when they are employed for molecular imaging using synchrotron and XFELs, compared to the original glass capillary-based GDVNs. Marmiroli et al. [37] presented a micromachining technique using X-ray lithography to engrave 60 µm thick channels into polymethyl-methacrylate (PMMA) slides, as demonstrated in Figure 4. They used finite element simulations to optimise the geometrical parameters in order to combine a micromixer with a free-standing liquid jet for time-resolved molecular studies at sub-0.1 ms resolution. Their microfluidic injectors were employed for synchrotron small-angle X-ray scattering (SAXS) measurements studying the formation of calcium carbonate from calcium chloride and sodium carbonate. The fastest recorded dynamics that they were able to track occurred on a timescale of just 75 µs.

**Figure 5:**
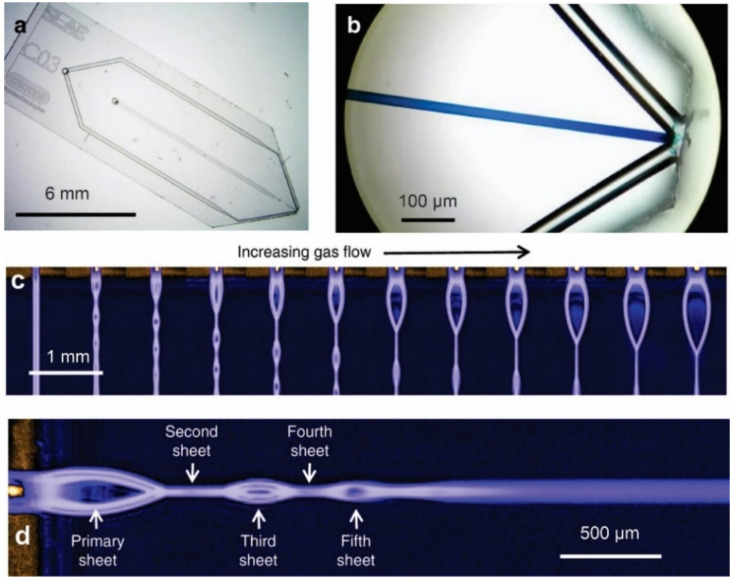
Microfluidic GDVN for ultrathin liquid sheet generation. (**a**) The microfluidic chip (6 × 19 mm) with gas and liquid ports incorporated, (**b**) liquid and gas microchannel can be distinguished via the introduction of blue dye into the liquid channel, (**c**) the jet regime varies as a function of gas pressure, (**d**) a detailed view of the alternating orthogonal liquid sheet structure (reproduced with permission from Reference [38]).

Koralek et al. [38] proposed a microfluidic glass chip fabricated using standard hard lithography to create sub-micron liquid sheets, as depicted in Figure 5. They performed optical, infrared, and X-ray spectroscopies to measure the thickness of the liquid sheet, which was found to range from approximately 20 nm to around 1 μm. The liquid sheet was stable for flow rates between 150 and 250 μL/min and a gas flow rate of around 100 SCCM. The nanometer-thick sheet could have transformative potential for applications in infrared, X-ray, electron spectroscopy studies.

**Figure 6:**
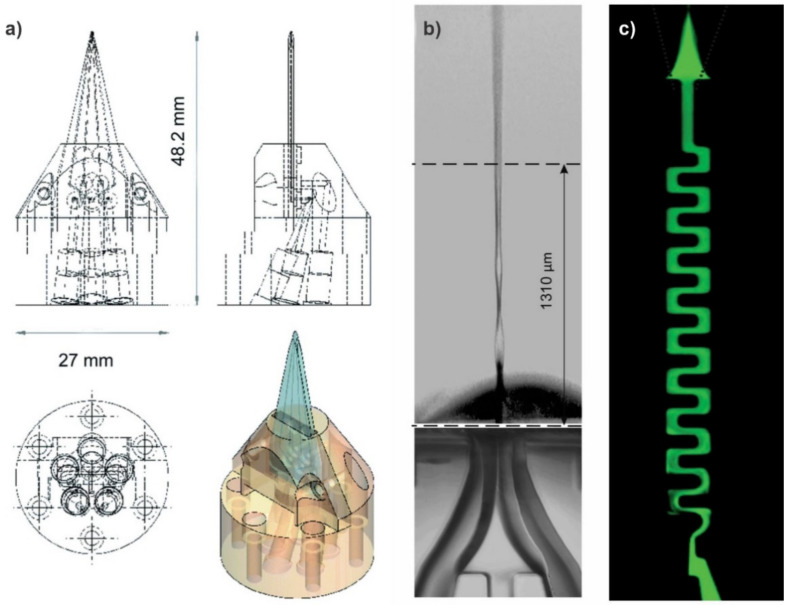
The SU8 on glass microfluidic mix-and-jet devices, (**a**) 3D schematics showing how the microfluidic chip is interfaced to tubing using a custom-made jig, (**b**) the ribbon regime created by the microfluidic mix-and-jet devices under gas flow rates ranging from 162 to 234 mg/min and liquid flow rates of 80 to 100 μL/min, (**c**) mixing of water and a diluted fluoresceine salt solution in the serpentine mixing component (reproduced with permission from Reference [39]).

Hejazian et al. [39,40,41] have proposed a novel SU8 on glass technique to fabricate mix-and-inject devices suitable for experiments at both the synchrotron and XFEL. The use of SU8 for the fabrication of the microchannels provides high chemical inertness and X-ray stability, which is further supported by a glass body to increase the mechanical rigidity making it suitable for enduring high pressures. The microchannels were made using high-resolution photolithography, which offers reproducibility and facilitates the fabrication of serpentine-shaped mixer microchannel structures. The integration of a planar passive micromixer demonstrated a superior mixing performance compared to a straight channel micromixer. Schematics of the 3D design of the jig, the liquid jetting, and the mixing component are demonstrated in Figure 6. Furthermore, they observed three distinct jetting regimes, including the ultrathin liquid sheets reported by Koralek et al. [38], which are achievable by only adjusting the operating conditions with a single device.

**Figure 7:**
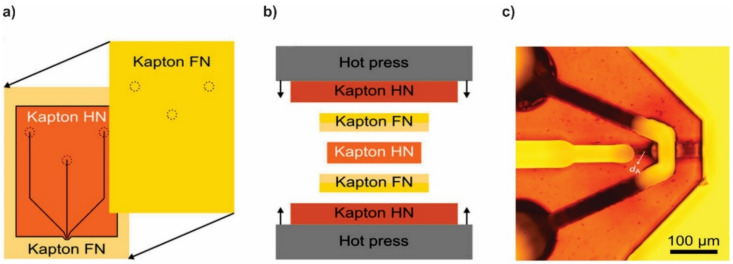
Schematic of the fabrication process of Kapton^®^ GDVN devices. (**a**) Alignment and hot embossing bonding of the Kapton^®^ foils, (**b**) stacking order of the bonding procedure, (**c**) microscopic image of the finished GDVN device showing gas and liquid microchannels (reproduced with permission from Reference [42]).

Vakili et al. [42] presented a prototyping technique based on laser ablation of Kapton^®^ polyimide foils for the fabrication of a microfluidic chip GDVN, shown in Figure 7. Kapton^®^ foils of 125 μm thickness were micromachined using a 193 nm argon fluoride (ArF) excimer laser and bonded to each other using hot embossing. The use of Kapton^®^ sheets has the advantages of having high chemical inertness and x-ray transparency which makes these devices ideal for serial crystallography experiments at synchrotrons and XFELs as well as SAXS measurements at these facilities.

### 3.3. Three-Dimensional Printed Microfluidic Devices

The fabrication processes discussed above typically involve time-consuming manual steps and have only limited capability for making complex true 3D micro-features. The 3D-printing fabrication technique has recently gained attention as a fully digital and automated rapid-prototyping method for producing small batches of customised microfluidic devices [43,44]. The technique also reduces assembly work due to the capability of printing chip holders and the chip-to-tubing connections [45].

Despite the current challenges in microfluidic 3D printing [11], for example, the comparatively low throughput, there have been numerous successful reports on the utilisation of submicron resolution 2-photon polymerisation (2PP) 3D printing techniques and using IP-S resist (Nanoscribe GmbH, Karlsruhe, Germany) printing material for the fabrication of mix-and-inject devices. Nelson et al. [46] introduced a 3D printed GDVN sample delivery device for time-resolved studies using an XFEL. They implemented a submicron resolution 2PP 3D printing technique to fabricate nozzle tips, which were glued to gas and liquid capillaries. The off-axis jetting of their first device was corrected by adjusting the design of the nozzle tip, characterised using X-ray tomography, achieving a straight jet. The 3D printing method was able to overcome the geometrical constraints of conventional fabrication methods, and their device was able to achieve stable jetting with a gas pressure lower than for glass GDVNs.

Galinis et al. [47] introduced 3D printed nozzles to create a stable thin liquid sheet jet in a vacuum, using high resolution (0.2 µm) direct two-photon laser writing. They used a custom-made plate holder for batch printing of the nozzles and the average printing time for each nozzle was around 2 h. The devices could withstand pressures of up to 8 bars and achieve a jet thickness within the range of 1.02–4.58 µm at 9.1 mL/min under both vacuum and normal atmospheric conditions. Wiedorn et al. [48] used the 3D printed nozzle design first reported in Nelson et al. [46] for high-resolution structure determination of hen egg-white lysozyme (HEWL) microcrystals (6–8 μm in diameter) using megahertz serial femtosecond crystallography (SFX) at the SPB/SFX beamline at the European XFEL. The 3D printed devices were able to deliver the sample using high-speed liquid jets with a 1.8 μm diameter at speeds of between 50 and 100 m/s to match the megahertz repetition rate, which is equivalent to a total of 150–1200 pulses per second. They found that the high-speed jet speeds produced by the 3D printed sample delivery device combined with the megahertz beamline could significantly reduce sample consumption and the data acquisition time.

Bohne et al. [49] have reported on a hybrid fabrication method consisting of 2PP 3D printing the nozzle head onto a 2D microfluidic silicon-glass chip fabricated via lithography. The method omits the assembly steps for connecting the nozzle tip to the liquid sample and gas channels, which previously required gluing of the capillaries to the nozzle tip. The device was capable of generating stable jets under atmospheric and vacuum conditions, with a 1.5 μm diameter at a liquid flow rate of 1.5 μL/min, and a more than 20 μm diameter at a flow rate of 100 μL/min. The hybrid method allowed for integrating multiple microfluidic components on a single chip to make custom-designed sample delivery devices to suit a particular sample’s characteristics.

Nazari et al. [50] employed 2PP 3D printing with an IP-S material to fabricate a GDVN nozzle which had an asymmetric design. Their method was able to achieve submicron resolution printing with a printing time of between 35 min to a few hours. The device could establish stable jets with speeds greater than 170 m/s, which is suitable for MHz XFEL experiments. They systematically characterised the liquid jets produced by the device and reported the jet diameter, length, speed, and Weber number as a function of the gas sheath flow rate by using a dual-pulsed nanosecond image acquisition and analysis method.

**Figure 8:**
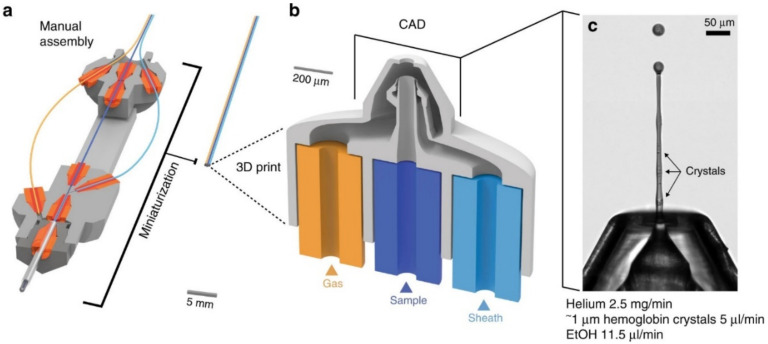
Three-dimensional printed double-flow-focusing GDVN. (**a**) The design assembly consisting of inserting three glass capillaries into a machined 10 cm long aluminium body, (**b**) 3D schematics of the gas orifice with three capillary inlets for gas, liquid sample, and sheath flow, (**c**) the 3D printed nozzle in operation jetting a solution containing 3 μm Hemoglobin crystals (reproduced with permission from [51]).

Knoska et al. [51] reported an optimised 2-photon stereolithography 3D printing technique achieved by adjusting the print resolution during fabrication to reduce the printing time for mix-and-inject sample delivery devices to minutes. The designed assembly and the liquid jet created by the device are demonstrated in Figure 8. The devices could achieve submicron jets with jet speeds higher than 200 m/s, suitable for megahertz time-resolved structural biology studies at XFELs. They also fabricated and tested a double-orifice nozzle for creating narrower jets with a reduced sample consumption for liquid jet samples. They introduced the X-ray microtomography technique for the characterisation of their 3D millisecond mixer component of the devices. Their 3D integrated micromixer consisted of a series of 180° turn helical elements that facilitate high mixing efficiencies, minimising inertial forces to avoid damaging the microcrystals in the liquid sample whilst maintaining a constant cross-section to prevent blockage of the device. The fabrication methods and their advantages and limitations that were discussed in Section 3 are summarised in Table 1.

## 4. Characterisation Techniques

The fabricated devices require lab testing and calibration before being implemented as sample delivery devices for applications such as molecular imaging at XFEL facilities. In this section, we summarise the standard methods that were used for the characterisation of mixing and jetting within integrated microfluidic mix-and-jet sample delivery devices.

### 4.1. Jetting Analysis

The analysis of jetting is mostly conducted through microscopic imaging of the microjet to map the stable and unstable regions as a function of the operating parameters, e.g., gas pressure and liquid flow rate. Vega et al. [21] used a Complementary Metal-Oxide-Semiconductor (CMOS) high-speed video camera (Photonfocus MV-D1024-160F, Photonfocus AG, Lachen, Switzerland) to image the fluid meniscus and the jet. The nozzle was illuminated using an optical fibre connected to a light source. An auxiliary charge-coupled devices (CCD) camera, positioned perpendicularly with respect to the CMOS camera, was used to assess the asymmetricity of the flow focusing by acquiring images of the liquid meniscus. The imaging setup was mounted on an optical table with a pneumatic antivibration isolation system for analysing both the stability of the liquid jets and the behaviour of the liquid meniscus.

Galinis et al. [47] measured the thickness of thin liquid sheet jet flows created by their 3D printed nozzles using white light interferometry using a 633 nm He–Ne laser. An optical fibre was used to guide the light into a spectrometer (OceanOptics HR4000, 200–1100 nm, Ocean Optics, Inc., Largo, FL, USA), and the peak values of the focused white light spectral interferograms were used to determine the absolute thickness and flatness of the liquid sheet under both atmospheric pressure and in vacuum conditions.

**Figure 9:**
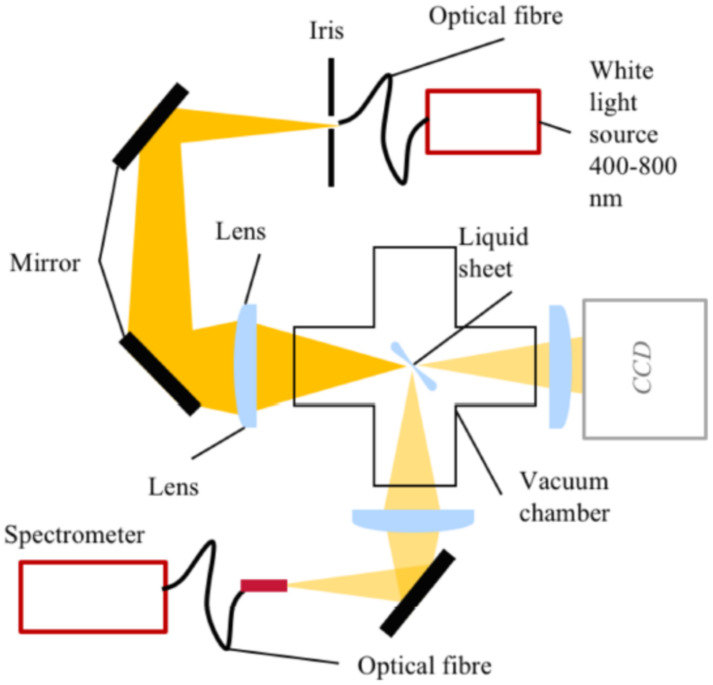
The layout of the testing station used to characterise the nozzle jetting performance under vacuum (reproduced with permission from Reference [30]).

Beyerlein et al. [30] examined jet stability and break up using a Photron FASTCAM SA4 camera (Photron PTY Ltd., Tokyo, Japan), with a frame rate of 500,000 frames per second and a shutter speed of 1 microsecond, for imaging. The high-speed camera was equipped with a 12× Ultra-Zoom motorised lens and a 10× objective lens to provide a resolution ranging from 0.3 to 3 μm/pixel to image both the jetting (with speeds of up to 20 m/s) and the 10 μm droplets, created after the jet break-up. Schematics of the experimental setup are depicted in Figure 9. Their fast-imaging setup takes advantage of an illumination source consisting of a Karl Storz xenon lamp generating a uniform background, which was coupled to a pulsed laser source to improve the time resolution. Bohne et al. [49] used the same setup as Beyerlein et al. [30] for measurements of liquid jets created by their 3D printed device under atmospheric pressure conditions. They used an environmental scanning electron microscope (SEM, EVO MA 25, Carl Zeiss AG, Oberkochen, Germany) to conduct the measurements under near-vacuum conditions of 100 Pa.

Knoska et al. [51] carried out submicron jet diameter measurements, with jet speeds of over 200 m/s, using a nanosecond double flash imaging. The laser light generated by the dual-pulse laser system (Nano S 50-20 PIV, Litron Lasers, Rugby, Warwickshire, England, UK) illuminated Rhodamine 6G dye (252433, Sigma–Aldrich, St. Louis, MO, USA) to determine the jet velocities directly from the recorded images. They measured jet diameters as small as 536 nm, at a liquid flow rate of 2.4 µL/min, and a gas flow rate of 22.5 mg/min, using their imaging setup.

### 4.2. Mixing Analysis

**Figure 10:**
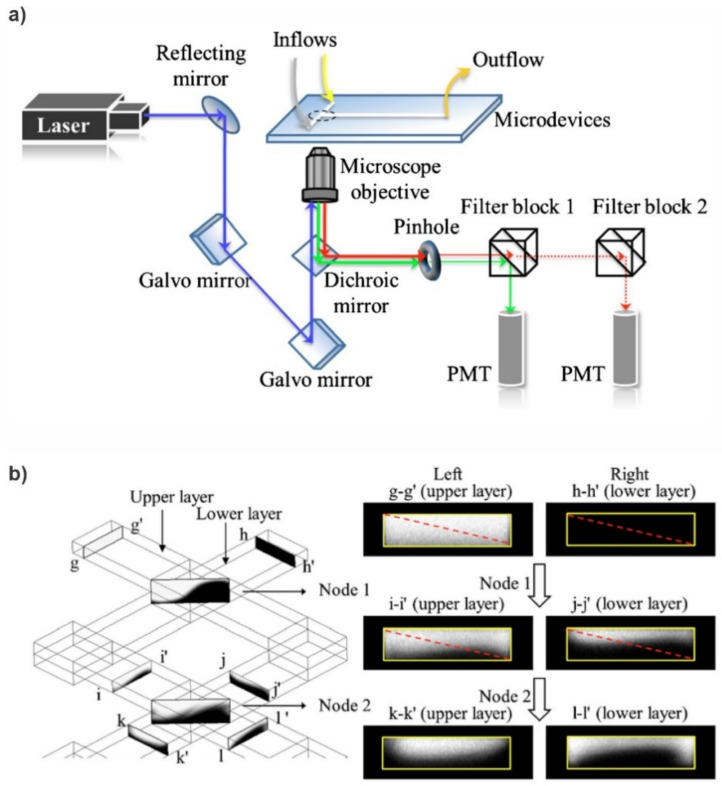
Confocal Fluorescence Microscope (CFM) for fluorescent imaging for mixing analysis. (**a**) Schematic diagram of the CFM system used by Fang et al., (**b**) schematics of the microfluidic mixer channel, and the cross-sectional fluorescent images depicting the progression of chaotic mixing along the mixer (reproduced with permission from Reference [52]).

The most common and straightforward method for investigating mixing in microfluidic devices is using a Confocal Fluorescence Microscope (CFM) for fluorescent imaging and then analysing the fluorescent intensity profiles to determine the mixing efficiency. Fang et al. [52] implemented a CFM for imaging and quantifying the 3D mixing patterns in microfluidic mixer devices, as shown in Figure 10. They used fluorescent intensity analysis to quantify the mixing efficiencies of the micro-mixers. In addition, they captured clear fluorescent images of the mixing patterns, which demonstrate flow advection and mass exchange. Inguva et al. [53] proposed a high-speed velocimetry technique for measuring fluid speeds of up to 10 m/s in microchannels to study chaotic mixing in microfluidic devices. They implemented a CFM equipped with a water-immersed Olympus UPLSAPO 60XW (Olympus Corp, Shinjuku City, Tokyo, Japan) objective to image a diffraction-limited confocal volume. Velocity profiles were established from analysing the data acquired at different depths within the micro-mixer. Their experimental method could measure fluid speeds with a 20% margin of error.

Xi et al. [54] used Optical Coherence Tomography (OCT) to examine and compare mixing efficiencies of three micro-mixers: a Y channel mixer, a 3D serpentine mixer, and a vortex mixer. They reported that significantly more accurate estimations of mixing efficiencies and flow velocity profiles could be achieved using the OCT method. The visual overlap of fluid flows when using confocal microscopy results in more accurate estimations of mixing efficiencies. Jiang et al. [55] used two-photon fluorescence lifetime imaging microscopy to visualise and study millisecond chaotic mixing dynamics inside microdroplets in an integrated droplet-based microfluidic serpentine mixer device. A flow rate of 1 µL/min was used for the sample streams, and 1.5 µL/min for the sheath flows with a 50 × 40 μm^2^ microchannel cross-section. Their fluorescent intensity analysis results show that the mixing efficiency inside the droplets can reach up to 80% after 18 ms.

Witkowski et al. [56] utilised micro-Particle Image Velocimetry (micro-PIV) to map the velocity profiles within passive micromixers. The images were acquired using an inverted laboratory microscope equipped with a 5.5-megapixel resolution camera. A laser light beam illuminates the fluorescent particles with a diameter of 1 µm, suspended in the carrier liquid flowing through the mixer microchannel. Yang et al. [57] proposed a method to simultaneously determine both the velocity and concentration profiles in microfluidic devices using micro-PIV and particle counting. They used a confocal fluorescence microscope for imaging the flow of microparticles inside the mixer microchannel. They used two distinct algorithms to track the displacement of microparticles for velocity profile determination whilst counting particles of different colours for resolving the concentration distribution.

**Figure 11:**
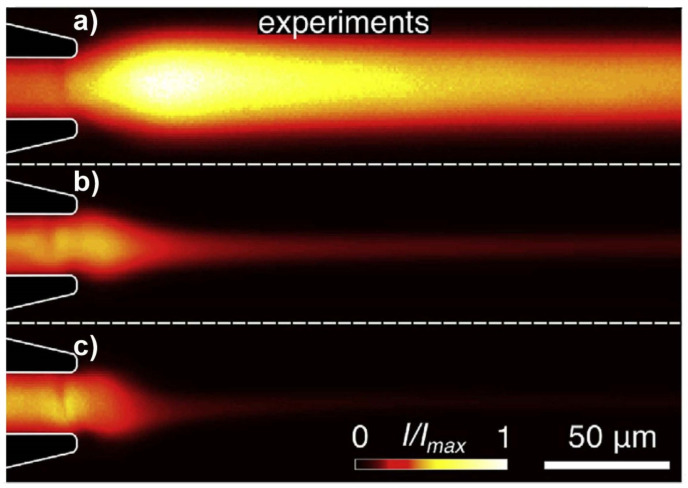
Experimental images of a hydrodynamic focusing mixer evaluated with the fluorescein-iodide quenching reaction technique for three sheath flow rates (*Q_sh_*) to sample flow rate (*Q_sa_*) ratios (*Q_sh_*/*Q_sa_*), (**a**) flow rate ratio of 100, (**b**) flow rate ratio of 1000, (**c**) flow rate ratio of 5000. At lower sheath to sample flow rate ratios, the sheath species diffuse into the sample stream (reprinted with permission from Reference [58]).

Huyke et al. [58] investigated both small time scales of mixing and homogenous residence times of a co-axial hydrodynamic focusing mixer using a fluorescein–iodide quenching reaction. A 50 mM fluoresceine sample was first hydrodynamically focused by a buffer sheath and then focused again by a 500 mM KI sheath, as shown in Figure 11. All solutions contained 20 mM Tris and 10 mM HeCl (Sigma–Aldrich, St. Louis, MO, USA) at a measured pH of 8. The mixing times for the fast-quenching fluorescent reaction were determined by fluorescent imaging using an inverted microscope equipped with a suitable illumination source. The mixing efficiencies were then quantified by analysing the fluorescent intensity of the acquired images. The mixing and jetting characterisation techniques that were discussed in Section 4 are summarised in Table 2 and Table 3.

## 5. Summary and Perspectives

The recent advances in molecular imaging techniques using cryo-EM, XFEL, and synchrotron facilities necessitates the precise and controlled delivery of mixed solutions. Microfluidic technology has shown promise in addressing the sample delivery needs for molecular imaging technology over recent decades. Here, we have reviewed the recent advances in the emerging field of integrated mix-and-jet microfluidic sample delivery devices.

We introduced the main parameters required for the design of these integrated devices. The nozzle component is mainly designed based on the GDVN principle and integrated into the microfluidic device to generate free-standing liquid jets. The primary dimensionless parameters to be considered for the nozzle design and characterisation of the jet are We and Re. Passive micromixers are commonly used to trigger biomolecular reactions, taking advantage of chaotic advection and rapid millisecond mixing. The main dimensionless parameters to be considered for the design of a passive mixing component are Re and *Pe*, whilst $\eta_{mixing}$ can characterise the mixing in the mixer microchannel. Additionally, we critically reviewed the techniques used for the fabrication of the mix-and-inject devices. Conventional capillary-based methods for the fabrication of the sample delivery devices are laborious and irreproducible, providing only limited versatility to integrate complex passive micromixers. Numerous techniques for the fabrication of chip-based microfluidic mix-and-inject devices were reported to replace the previous capillary-based techniques. Most of the chip-based planar methods enable the fabrication of rigid and chemically inert devices whilst taking advantage of the design freedom, high resolution, and reproducibility. Recently, 3D printed mix-and-jet microfluidic devices have shown great promise for XFEL single-particle imaging and SFX studies. The new technology facilitates fast and low-cost fabrication of fully 3D mixer and nozzle components that outperform both capillary and on-chip sample delivery devices. Furthermore, we summarised the standard experimental techniques used for the characterisation of both mixing and jetting. For these measurements, both high-speed optical imaging and fluorescent signal analysis were used.

Incorporating GDVN nozzles with microfluidics technology is still a new concept that will open up a host of new applications in many areas, especially in the biological and life sciences. Currently, most of the published references in this field are proof-of-concept of mix-and-inject experiments in which new device architectures and designs are often introduced. In the near future, we can expect to see more reports describing innovative designs and solutions to apply these devices to a range of different fields, including fundamental chemistry and physics, polymer fabrication, the study of the kinetics of nanoparticles, and biomolecular imaging.

## Figures and Tables

**Table 1:** Summary of fabrication methods (see Section 3) along with their pros and cons.

Fabrication Method	Pros	Cons
Co-axial capillary devices fabricated via glass extrusion [27,28]	High pressure and solution pH resistance and uses well-established fabrication methods.	Arduous manual intervention required during fabrication and assembly; poor reproducibility.
Co-axial capillary devices fabricated via ceramic micro-injection moulding [30]	Good reproducibility and reduced fabrication complexity compared to glass co-axial capillary devices.	Manual intervention required during fabrication, processing, and device assembly.
Microfluidic injector devices fabricated in PDMS [33,34,35,36]	Straight forward fabrication protocols, reproducible results, high spatial resolution.	Lack of mechanical stability and chemical inertness. Can only handle low pressures.
Deep X-Ray Lithography (DXRL) in PMMA [37]	Reproducible fabrication and high resolution.	Requires access to a synchrotron beamline; low PH resistance due to using PMMA.
Microfluidic glass chip fabrication using hard lithography [38]	High spatial resolution and reproducibility. Chemically and mechanically robust.	Costly manufacturing processes involving a high degree of complexity.
Microfluidic SU8 on glass lithographic fabrication [39,40,41]	Simple fabrication achieving high resolution combined with chemical and mechanical inertness and design flexibility.	Requires additional micromachining to produce the device inlet and outlet.
Laser ablation of Kapton^®^ polyimide fims [42]	High resolution, and high chemical and mechanical inertness.	Manual alignment required during fabrication employing laser micromachining.
Microfluidic devices fabricated via 3D nanoprinting [46,47,48,49,50,51]	Automated rapid-prototyping, high spatial resolution, and reproducibility possible.	Requires manual assembly and use of glass capillaries, limited flexibility in terms of geometry.

**Table 2:** Summary of the techniques used to characterise liquid jetting (see Section 4).

Method	Schematic	Comments
Complementary Metal-Oxide-Semiconductor (CMOS) high-speed video camera [21]	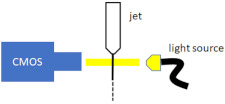	Used to measure the stability of the liquid jet and to study the behaviour of the liquid meniscus.
White-light interferometry [47]	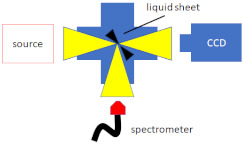	Measures the absolute thickness and ‘flatness’ of the liquid sheet under both atmospheric pressure and vacuum conditions.
High-speed microscopic imaging [30,49]	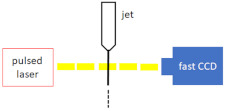	Used to study the liquid jet stability and the break up of the jet into microdroplets.
Nanosecond double flash imaging [51]	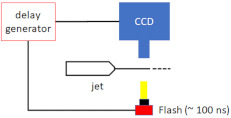	Used to determine the jet velocity and jet diameter.

**Table 3:** Summary of the techniques used to characterise microfluidic mixing (see Section 4).

Method	Schematic	Comments
Confocal Fluorescence Microscopy (CFM) [52]	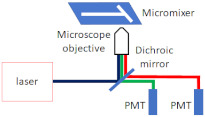	CFM is able to image and quantify the 3D mixing patterns on the microfluidic device.
High-speed velocimetry [53]	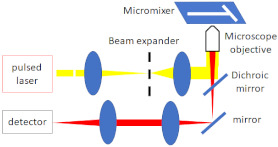	Applied to the study of chaotic mixing via measurements of the fluid velocity.
Optical Coherence Tomography (OCT) [54]	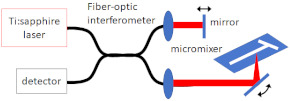	Enables an estimation of the 3D mixing efficiency.
Micro Particle Image Velocimetry (PIV) [55,56,57]	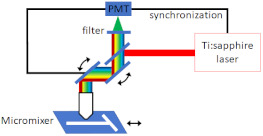	Can be used to map the velocity profiles within passive micromixers.
Fluorescein–iodide quenching reaction [58]	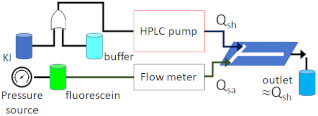	Enables measurement of the mixing times and mixing efficiencies

## Data Availability

The data that support the findings of this study are available from the corresponding author upon reasonable request.
